# MALAT1 is a prognostic factor in glioblastoma multiforme and induces chemoresistance to temozolomide through suppressing miR-203 and promoting thymidylate synthase expression

**DOI:** 10.18632/oncotarget.15199

**Published:** 2017-02-08

**Authors:** Wei Chen, Xin-Ke Xu, Jun-Liang Li, Kuan-Kei Kong, Hui Li, Cheng Chen, Jing He, Fangyu Wang, Ping Li, Xiao-Song Ge, Fang-Cheng Li

**Affiliations:** ^1^ Department of Neurosurgery, Guangzhou Women and Children's Medical Center, Guangzhou Medical University, Guangzhou 510623, China; ^2^ Department of Neurosurgery, Sun Yat-Sen Memorial Hospital, Sun Yat-Sen University, Guangzhou 510120, China; ^3^ State Key Laboratory of Virology, Wuhan Institute of Virology, Chinese Academy of Sciences, Wuhan, 430071, China; ^4^ Department of Respiratory, The First People's Hospital of Foshan, Sun Yat-Sen University, Guangdong Foshan 528000, China; ^5^ Department of Oncology, The Affiliated Hospital of Jiangnan University, Wuxi 214062, Jiangsu, China

**Keywords:** MALAT1, temozolomide, chemoresistance, miR-203, thymidylate synthase

## Abstract

Glioblastoma multiforme (GBM) is the most malignant brain tumor with limited therapeutic options. Temozolomide (TMZ) is a novel cytotoxic agent used as first-line chemotherapy for GBM, however, some individual cells can't be isolated for surgical resection and show treatment-resistance, thus inducing poor prognosis. By using the HiSeq sequencing and bioinformatics methods, we identified lncRNAs showing different expression levels in TMZ-resistant and non-resistant patients. RT-qPCR was then performed in tissues and serum samples, and lncRNA MALAT1 was finally identified to show considerable discriminating potential to identify responding patients from non-responding patients. Moreover, high serum MALAT1 expression was associated with poor chemoresponse and survival in GBM patients receiving TMZ treatment. Subsequently, the TMZ resistant cell lines were established, and the CCK8 assay showed that lncRNA MALAT1 knockdown significantly reversed TMZ resistance in GBM cells. The gain and loss-function experiments revealed that miR-203 was down-regulated by MALAT1 and this interaction has reciprocal effects. Besides, thymidylate synthase (TS) mRNA was identified as a direct target of miR-203. LncRNA MALAT1 inhibition re-sensitized TMZ resistant cells through up-regulating miR-203 and down-regulating TS expression. On the other hand, MALAT1 overexpression promoted resistance by suppressing miR-203 and promoting TS expression. In conclusion, our integrated approach demonstrates that enhanced expression of lncRNA MALAT1 confers a potent poor therapeutic efficacy and inhibition of MALAT1 levels could be a future direction to develop a novel therapeutic strategy to overcome TMZ resistance in GBM patients.

## INTRODUCTION

Glioblastoma multiforme (GBM) is one of the most common and aggressive form of primary brain tumors. It represents 12–15% of intracranial tumors and 60–75% of astrocytic tumors with widespread invasion in brain, poor differentiation, destruction of normal brain tissue and resistance to traditional therapeutic approaches [1–3]. Currently, temozolomide (TMZ)-based chemotherapy after surgical resection is one of the most frequently used therapeutic strategies. However, a large proportion of patients receiving chemotherapy finally become metastatic and chemoresistant, and this has been a key barrier to the efficacy of GBM treatment [4]. Previous study indicated that more than half of the patients were detected with overexpression of O-6-methylguanine-DNA methyltransferase (MGMT), which further leads to the failure of TMZ-based treatment [5]. Thus, the lack of effective therapies for the progression of GBM urges studies on the molecular etiology and identification of novel therapeutic targets including non-coding RNAs.

Long noncoding RNAs (lncRNAs) are defined as transcripts > 200 nucleotides in length and are transcribed but non-translated noncoding RNAs in human genome [6]. Altered expression of several lncRNAs has recently been attributed to pathogenesis of some malignant neoplasia, including GBM [7]. Numerous reports have demonstrated that its misregulation has a functional role in various types of cancers [8, 9]. MicroRNAs (miRNAs) are a class of short, endogenous, single-stranded RNAs that regulate gene expression. Until now, the role of lncRNA has not been well studied especially on the whole genome level, such as by using the high throughput sequencing method [10, 11]. However, the association of specific lncRNA or miRNA with drug resistance in GBM cells is largely unknown.

Thymidylate synthase is a critical regulator during cell proliferation and cell cycle progression. It catalyzes the reductive methylation of deoxyuridine monophosphate to deoxythymidine monophosphate with the reduced folate 5,10-methylenetetrahydrofolate as the methyl donor [12]. It is known that most of the intracellular source of thymidylate comes from this reaction procedure, and TS has been identified as a main target of various of chemo-therapeutics [13]. Hence, researchers have paid more and more attention to find a more effective way to overcome chemoresistance. It is well established that lncRNAs can participate in cancer progression through interacting with miRNAs [14]. This interaction may also play important function during GBM chemoresistance.

In this study, we conducted high-throughput Hiseq sequencing followed by reverse transcription quantitative real-time PCR (RT-qPCR) assays to test the hypothesis that specific lncRNAs can be useful in predicting chemo-response with the hope that such findings may guide therapeutic choice. Our date showed that lncRNA MALAT1 expression was significantly up-regulated in primary tissues and serum samples from GBM patients showing resistance to TMZ based treatment. Besides, the subsequent function assay revealed that lncRNA MALAT1 promoted TMZ resistance through suppressing miR-203 and activating TS function.

## RESULTS

### Identification of candidate lncRNAs by high-throughput Hiseq sequencing

**Figure 1:**
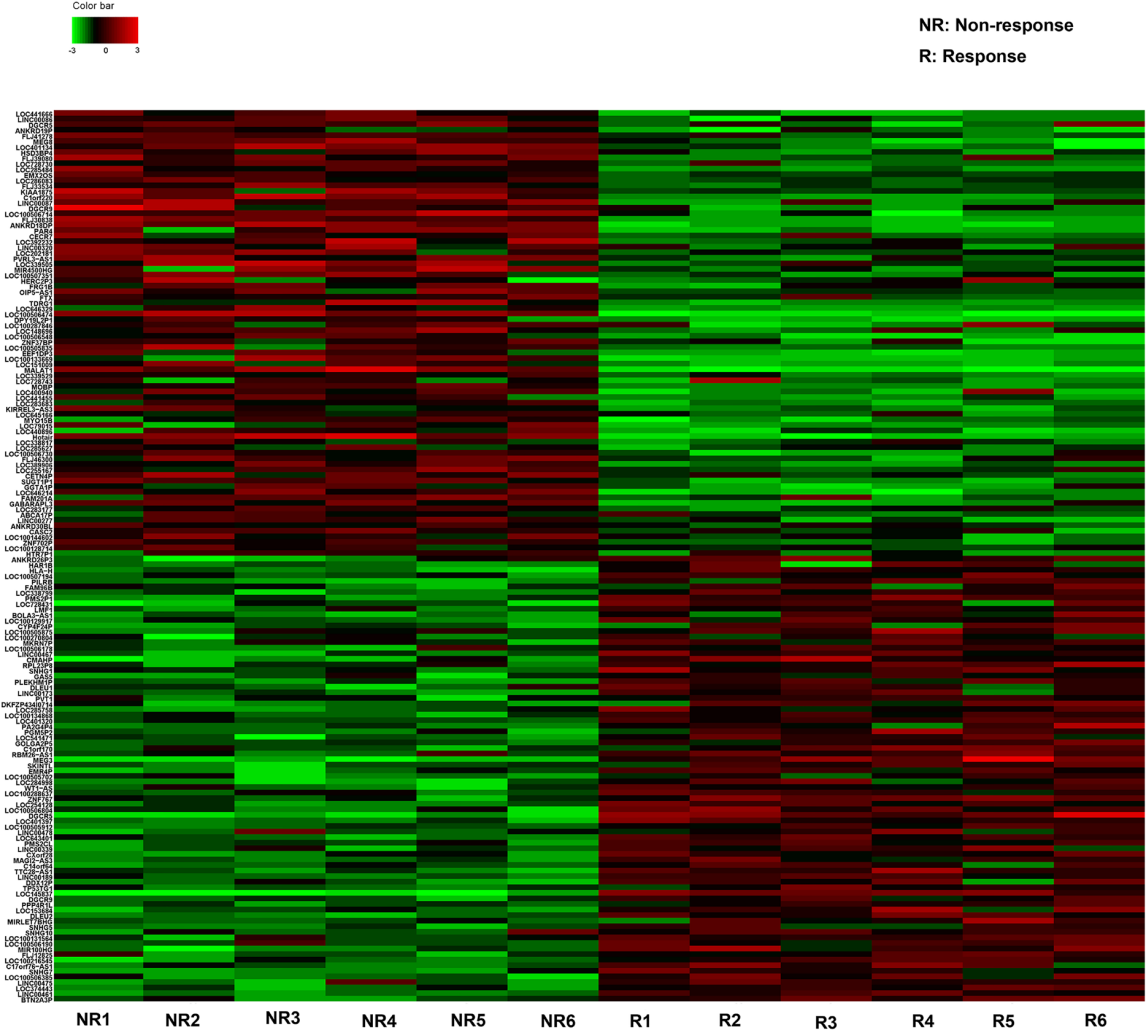
The heat map shows expression of the 160 lncRNAs most up- or down-regulated in GBM responding compared with non-responding patients to TMZ treatment The top 80 lncRNAs up- and down-regulated in GBM are shown in the top and bottom halves, respectively. The heat map was generated with an R package using normalization across rows (tissues).

The Hiseq sequencing with six tissue samples pooled from GBM patient showing response and six from patients showing no response to TMZ treatment were conducted. Our primary date showd that 637 lncRNAs expressed more than 2-fold change between responding and non-responding patients. Among these potential differently expressed lncRNAs, we concentrated on the top 80 most up- and down-regulated lncRNAs that were differentially expressed between resistant and non-resistant GBM patients (Figure 1). The expression of a lncRNA was considered altered only if at least 50 copies were detected by Hiseq sequencing, together with greater than five-fold change in its expression level between the responding and non-responding patients. Finally, we chose six lncRNAs on a basis of the Hiseq analysis (Table 1) and another four lncRNAs which had shown functional role during GBM chemoresistance [7]. Thus, 10 lncRNAs were selected as candidates for further testing via RT-qPCR.

**Table 1:** Candidate lncRNAs selected on a basis of the Hiseq analysis

Seqname	Location	Regulation (NR vs R)	Fold change	*P* value
MALAT1	Chr11q13.1	Up	89.2317621	0.00003751
LOC100506474	Chr2p24.3	Up	47.6729041	0.00113209
HOTAIR	Chr12q13.13	Up	31.2389160	0.00572906
LOC145837	Chr15q23	Down	43.8710538	0.00059024
DGCR5	Chr22q11.21	Down	34.3218649	0.00147631
MEG3	Chr14q32.2	Down	26.3691684	0.01782194

NR: Non-response; R: Response.

### MALAT1 is down-regulated in GBM patients showing response to TMZ treatment by RT-qPCR

**Figure 2:**
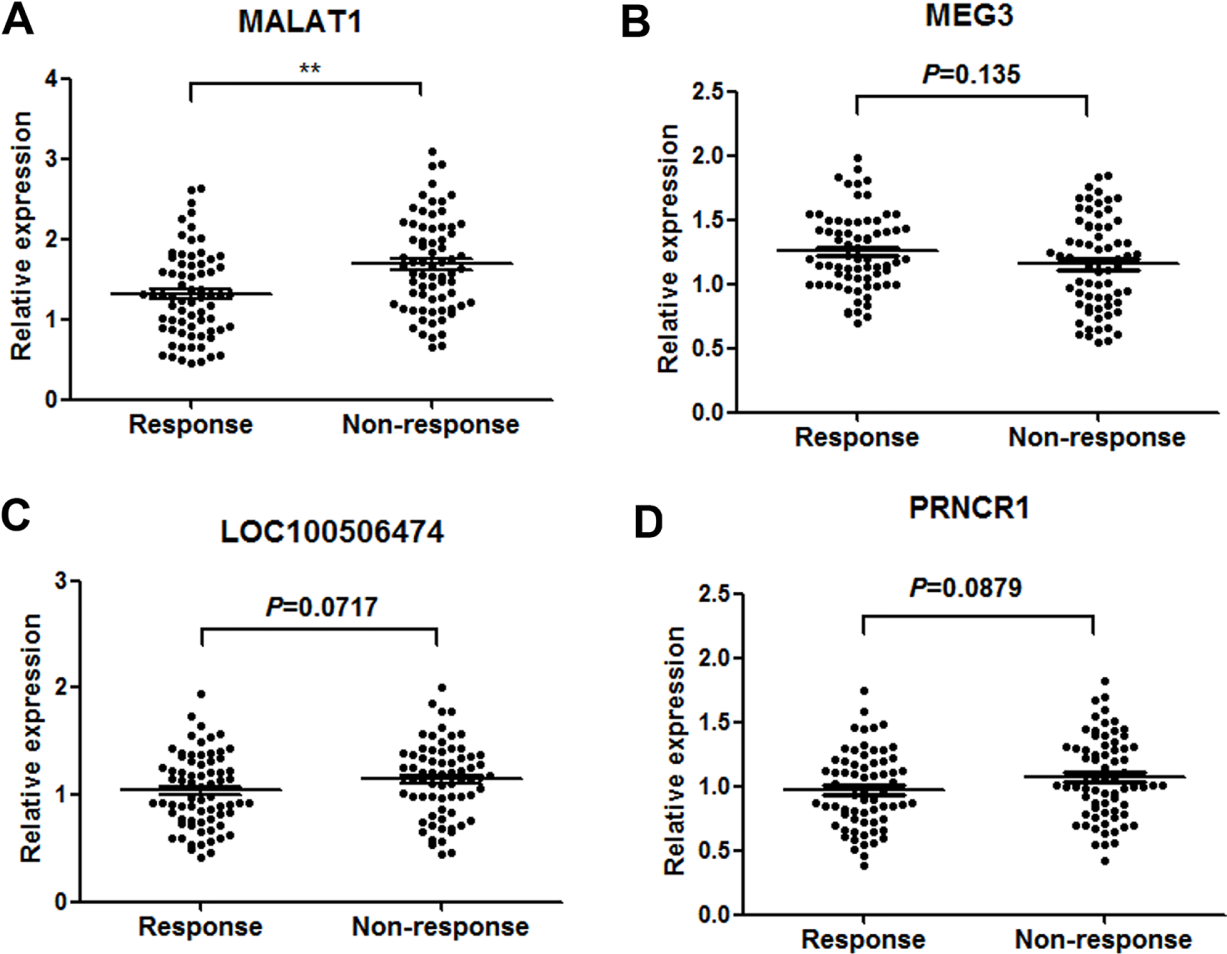
Concentrations of four identified serum lncRNAs in GBM patients showing response (*n* = 70) and non-response (*n* = 70) to TMZ therapy using RT-qPCR assay in validation set (**A**–**D**). **P < 0.01.

Firstly, RT-qPCR was performed to verify the 10 lncRNAs using 90 GBM tissues from patients showing response to TMZ and 90 from patients showing no response. Among these, four lncRNAs (MALAT-1, LOC100506474, MEG3 and PRNCR1) were found significantly dysregulated in responding tissues compared with non-responding tissues (Table 2). Then, the four lncRNAs were validated in another group of 140 serum samples from 70 GBM patients showing response and 70 showing no response. Among the four candidate lncRNAs, only MALAT1 was significantly desregulated with a dramatically suppressed expression in responding patients compared with non-responding patients (Figure 2A–2D). Thus, we focus on the role of MALAT1 in GBM chemotherapy.

**Table 2:** Expression of 10 candidate lncRNAs in GBM patients showing response or non-response to TMZ treatment [median (interquartile range)]

LncRNA	Response	Non-response	*P* value
MALAT1	1.29 (0.41–2.78)	2.37 (1.29–3.38)	< 0.01
LOC100506474	0.77 (0.45–1.93)	1.36 (0.42–2.06)	< 0.01
HOTAIR	1.07 (0.31–2.49)	1.54 (0.54–3.16)	0.29
PRNCR1	0.61 (0.32–1.71)	1.35 (0.35–1.98)	< 0.05
PCGEM1	1.01 (0.41–2.05)	1.29 (0.93–2.57)	0.11
LOC145837	0.89 (0.34–2.75)	0.67 (0.25–1.65)	0.08
DGCR5	1.12 (0.45–2.48)	0.94 (0.45–1.48)	0.07
MEG3	1.63 (0.43–2.37)	0.83 (0.27–1.74)	< 0.05
AGAP2-AS1	0.82 (0.23–1.67)	1.16 (0.44–2.82)	0.08
H19	1.04 (0.48–1.68)	1.12 (0.61–2.28)	0.34

### High serum MALAT1 expression was correlated with poor response to TMZ treatment

**Figure 3:**
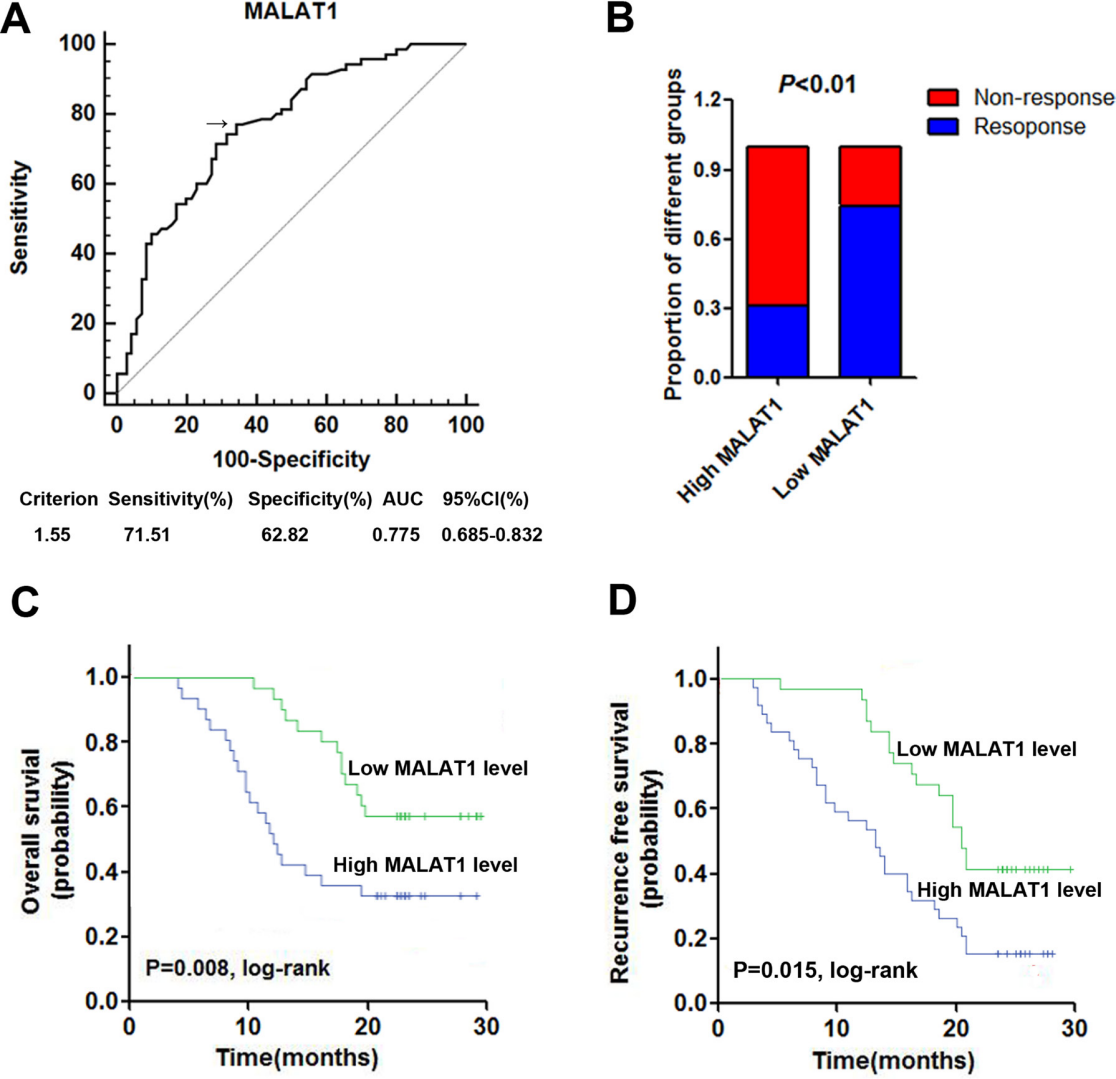
High serum MALAT1 expression was associated with poor response to TMZ treatment in GBM patients (**A**) ROC curves for differentiating the responding patients from non-responding patients of GBM using MALAT1 in validation set; (**B**) The proportion of patients that responded to TMZ treatment was significantly higher in the low MALAT1 expressing group than in the high MALAT1 expressing group; (**C**–**D**) Kaplan-Meier curves for OS (C) and RFS (D) according to serum levels of MALAT1 in GBM patients in validation set.

Firstly, receiver operator characteristic (ROC) curve was drawn to investigate the potential diagnostic value of serum MALAT1 in differentiating the chemoresponse status in GBM patients. The area under the curve (AUC) was 0.764, with the diagnostic sensitivity and specificity reaching 77.1% and 65.7%, respectively (Figure 3A). Under the stratification criteria (1.33) established by the ROC curve, the number of patients that responded to TMZ treatment was significantly higher in the low MALAT1 expressing group than in the high MALAT1 expressing group (Figure 3B). Additionally, Kaplan–Meier survival analysis showed that high expression of serum MALAT1 was correlated with poor OS and RFS (Figure 3C and 3D). Furthermore, we performed Cox regression univariate/mutivariate analysis to identify whether MALAT1 or other clinical parameter was an independent indicator for OS of GBM patients who received TMZ chemotherapy. The results indicated that serum MALAT1 expression level and WHO grade maintained their significance as independent prognostic factors for OS of GBM patients receiving TMZ treatment (Table 3).

**Table 3:** Univariate and multivariate Cox proportional hazards regression model analysis of OS in patients with GBM in validation set

Characteristics	Univariate analysis			Multivariate analysis		
	HR	95% CI	*P* value	HR	95%CI	*P* value
Gender	1.032	0.677–3.029	0.399			
Age	1.477	0.734–3.467	0.242			
Tumor size	1.445	0.615–2.851	0.327			
KPS	2.156	1.041–3.662	0.034	2.361	1.307–4.225	0.057
WHO grade	3.678	1.502–7.486	0.011	3.573	1.489–7.492	0.023
Serum MALAT1	2.318	1.203–4.875	0.009	2.553	1.223–5.201	0.008

KPS: Karnofsky Performance Status.

### Knockdown of MALAT1 reverses chemoresistance in TMZ resistant cells

**Figure 4:**
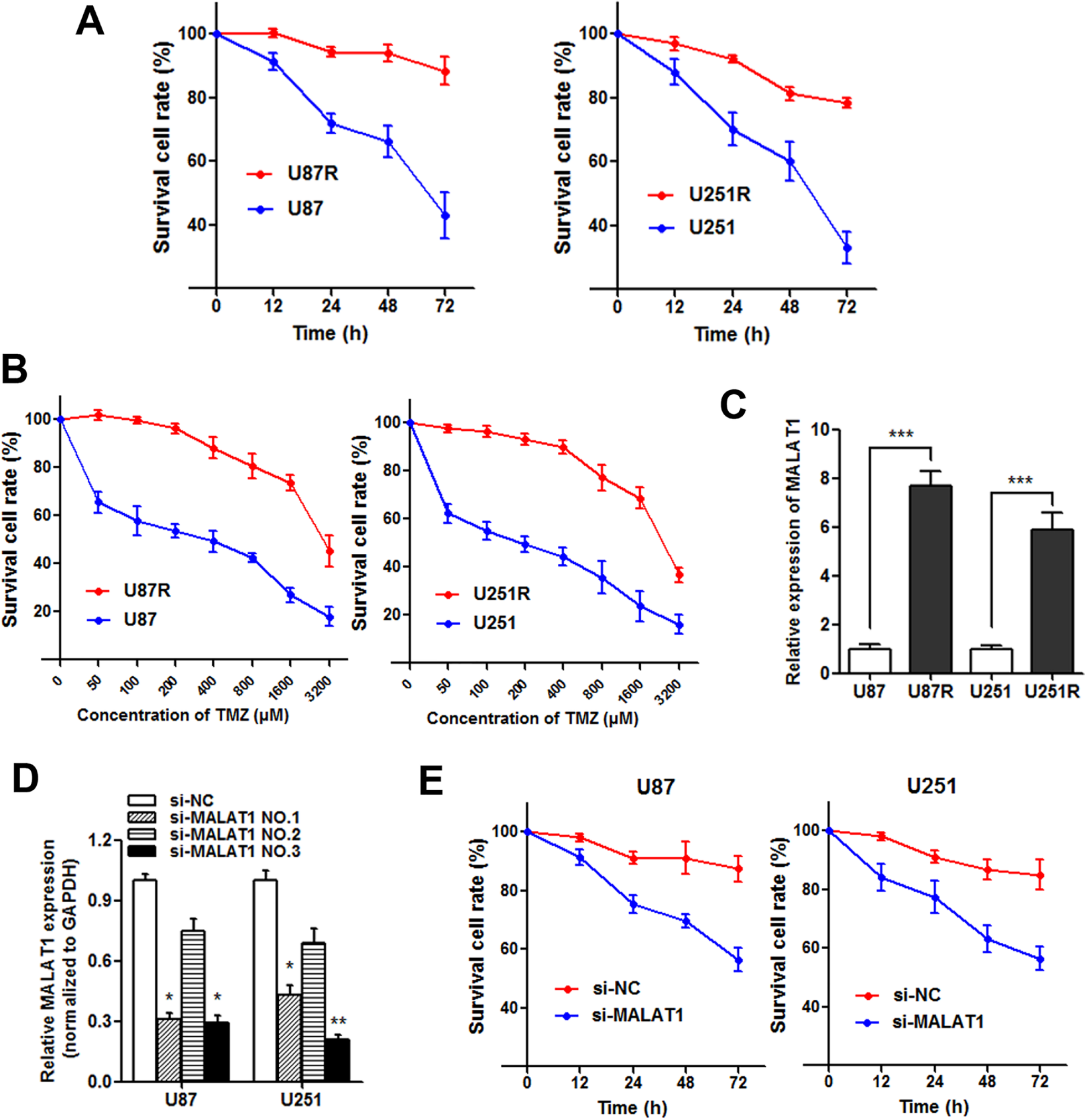
Knockdown of MALAT1 reverses chemoresistance in TMZ resistant cells (**A**) Both U87R and U251R cells showed elevated cell viability compared with U87 and U251 parental cells when incubated with culture medium containing 325 μM concentration of TMZ; (**B**) The concentration -effect curve indicated that the IC_50_ values of TMZ on U87R and U251R cells were significant higher than that on U87 and U251 parental cells; (**C**) The expression of MALAT1 in U87R and U251R cells were significantly higher than that in U87 and U251 parental cells, respectively; (**D**) Glioma cells were transfected with 100 nmol/L of the indicated siRNA. Forty-eight hours after transfection, RT-qPCR was performed using the primers for MALAT1; (**E**) Cell viability was significantly damaged when MALAT1 was silenced in U87R and U251R cells compared with control. *P < 0.05, **P < 0.01, ***P < 0.001.

TMZ-resistant cell lines U87R and U251R were established as described in Materials and methods. The established TMZ-resistant cells were maintained and exposed to 325 μM TMZ unless otherwise indicated. As shown in Figure 4A, both U87R and U251R showed elevated cell viability compared with U87 and U251 parental cells when incubated with culture medium containing 325 μM concentration of TMZ. On the other hand, the concentration -effect curve indicated that the IC_50_ of TMZ on U87R was 2478 μM, while the IC_50_ of TMZ on U87 was 277.5 μM, which means that the U87R was 8.93 times the ability of TMZ resistance of U87. Similarly, the U251R was 8.14 times the ability of TMZ resistance of U251 (1936μM/237.8μM, Figure 4B). Subsequently, we detected the MALAT1 expression level in U87R and U251R cells, and found that MALAT1 was significantly increased in U87R and U251R compared with the parental cells, respectively (Figure 4C). To assess the role of MALAT1 in GBM resistance, we silenced MALAT1 expression in GBM cell lines by small interfering RNA (Figure 4D) and si-MALAT1 (NO.3) used for silencing MALAT1. Our results showed that cell viability was significantly damaged when MALAT1 was silenced in U87R and U251R cells incubated with TMZ (Figure 4E). This suggests that MALAT1 knockdown partially reverses the TMZ resistance in GBM cells.

### MALAT1 negatively regulates miR-203 expression in GBM cells

**Figure 5:**
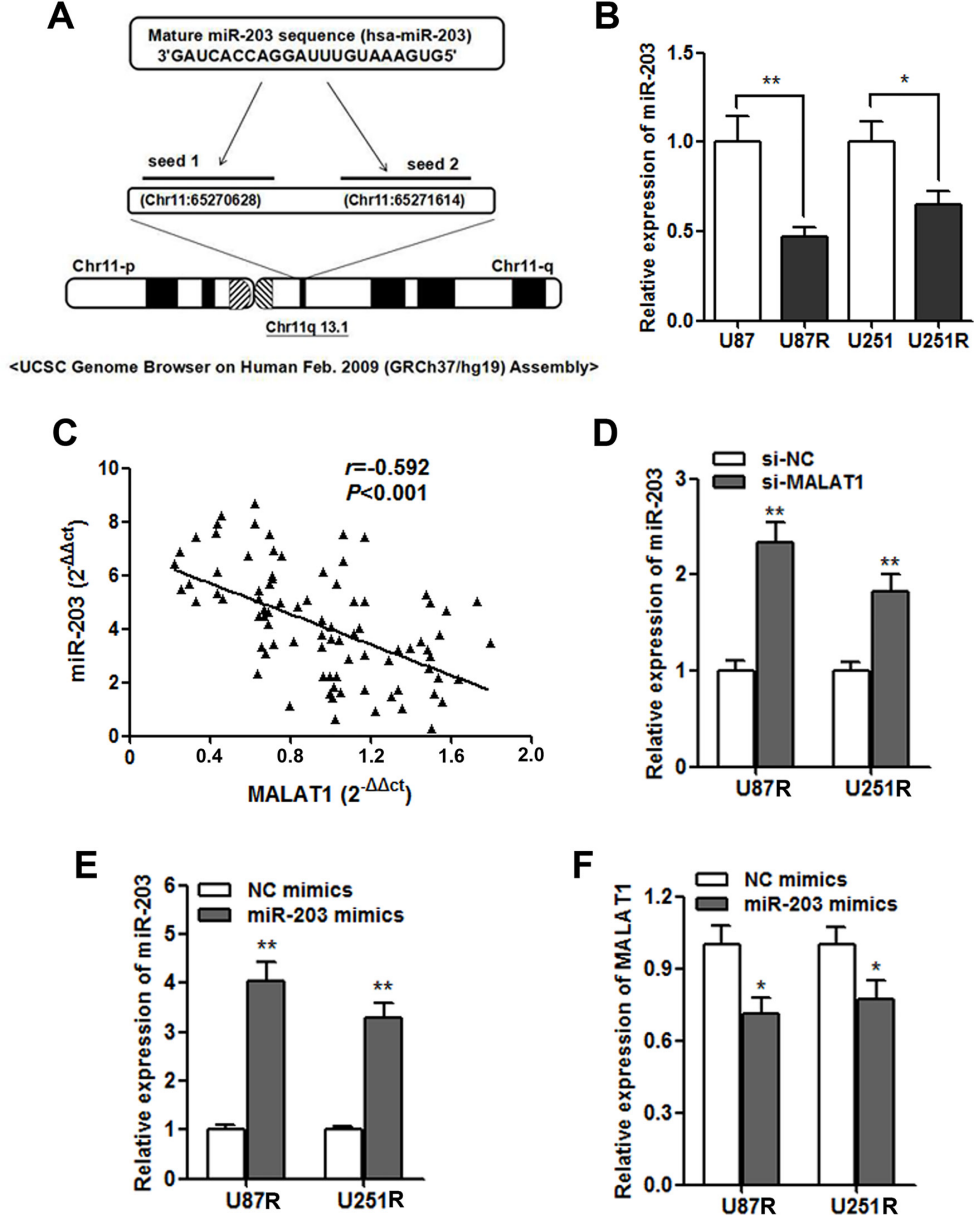
MALAT1 negatively regulates miR-203 expression in GBM cells (**A**) Representation of the miR-203 binding site in MALAT1 based on miRcode (http://www.mircode.org/mircode/); (**B**) RT-qPCR results showed that miR-203 expression level was significantly down-regulated in U87R and U251R cells when compared with U87 and U251 parental cells, respectively; (**C**) Negative correlation was found between the miR-203 levels and the MALAT levels in 140 serum samples of GBM patients on validation set; (**D**) miR-203 expression was significantly up-regulated in U87R and U251R cells transfected with si-MALAT1 when compared with cell transfected with si-NC; (**E**) The transfection of miR-203 mimics significantly promoted miR-203 expression in U87R and U251R cells; (**F**) MALAT1 expression was down-regulated by transfection of miR-203 mimics in U87R and U251R cells. *P < 0.05, **P < 0.01.

We further investigated the potential regulatory mechanism of MALAT1 on GBM chemoresistance. By using miRcode (http://www.mircode.org/mircode) [15], we identified two miR-203 MALAT1 binding sites (Figure 5A). Additionally, miR-203 was found to play important role during chemoresistance in cancer [16]. The RT-qPCR showed that miR-203 was dramatically down-regulated in the TMZ resistant cells (Figure 5B), and a significant negative correlation was also found between MALAT1 and miR-203 expression in the validation set (Figure 5C). Moreover, miR-203 was significantly up-regulated after MALAT1 was silenced by si-MALAT1 transfection in GBM cells (Figure 5D). On the other hand, miR-203 also significantly decreased MALAT1 expression (Figure 5E and 5F).

### MiR-203 re-sensitizes TMZ resistant cells through directly targeting TS

**Figure 6:**
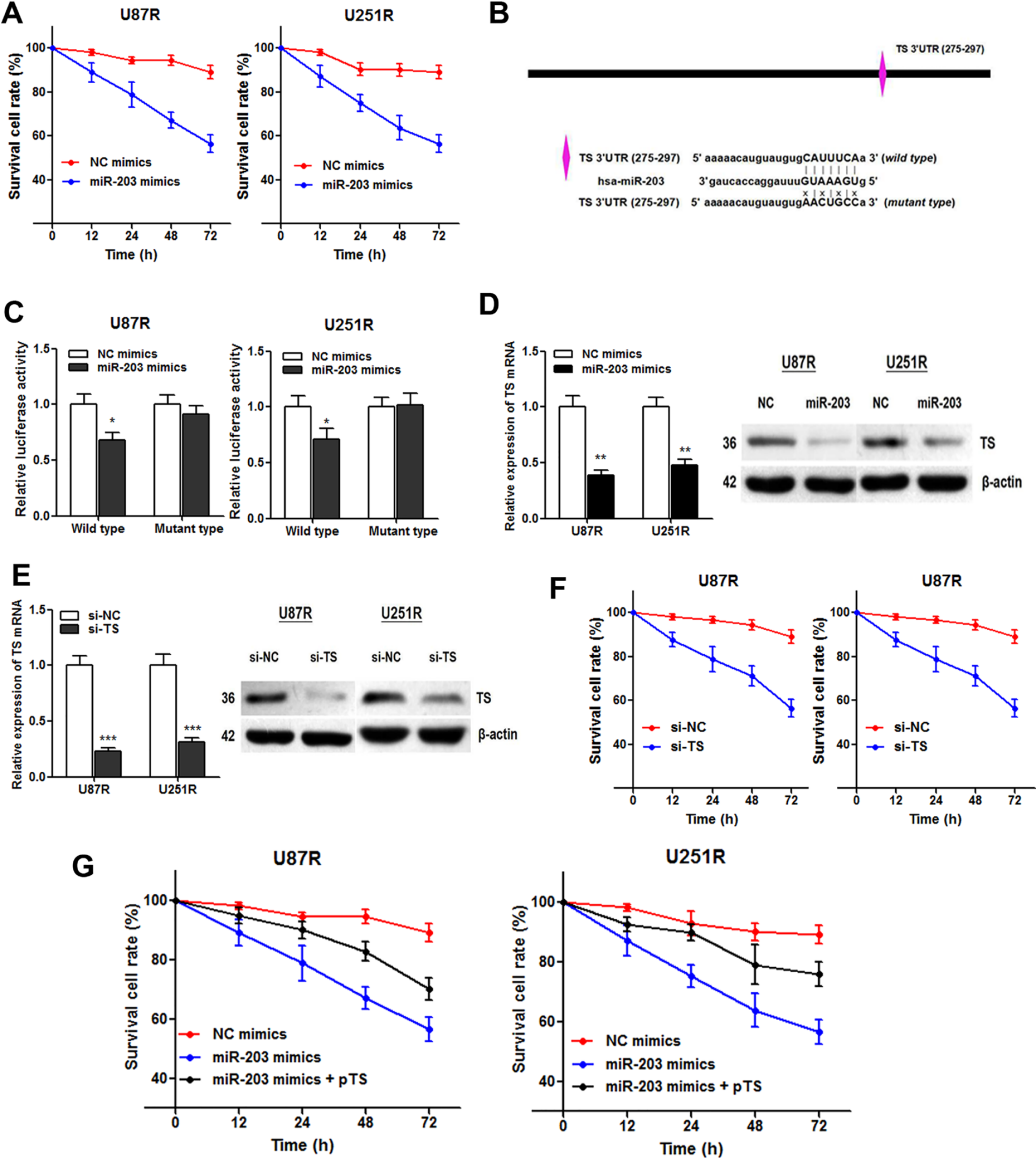
miR-203 re-sensitizes TMZ resistant cells through directly targeting TS (**A**) CCK8 assay showed that miR-203 overexpression enhanced TMZ induced cytotoxicity; (**B**) Illustration of the putative predicted miR-203 binding site in the TS mRNA 3′UTR region, and the mutation within the binding site was generated; (**C**) miR-203 targets the wild-type but not the mutant 3′UTR of TS mRNA in U87R and U251R cells; (**D**) miR-203 transfection significantly suppressed TS mRNA (left panel) and protein expression level (right panel) in both TMZ cell lines; (**E**) TS-specific siRNAs were generated and resulted in a marked decrease in TS mRNA (left panel) and protein level (right panel); (**F**) TS knockdown suppressed the survival cell rate of the U87R and U251R cells compared with the control; (**G**) Overexpression of TS with pTS dramatically abrogated the miR-203 induced re-sensitization to TMZ treatment in U87R and U251R cells. *P < 0.05, **P < 0.01, ***P < 0.001.

Based on the above results, we wondered whether miR-203 participated in the TMZ resistance in GBM cells. The cell viability assay showed that miR-203 transfection significantly enhanced the TMZ induced cell cytotoxicity (Figure 6A). Then, we used Targetscan and miRanda to predict miR-203 target genes, and a potential binding site was found in 3′UTR of TS mRNA (Figure 6B). We then constructed a reporter vector containing the wild and mutant TS 3′UTR downstream of the luciferase coding gene. As expected, luciferase activities of cells transfected with wild type TS reporter were significantly lower after transfection of miR-203, whereas those with mutant type TS reporter showed no significant difference (Figure 6C). Moreover, both TS mRNA and protein expression level was significantly suppressed by miR-203 in GBM cell lines, revealing that TS mRNA acts as a downstream target of miR-203 in GBM (Figure 6D).

Subsequently, we tested if TS is responsible for the miR-203 induced re-sensitization to TMZ treatment. TS-specific siRNAs were generated (Figure 6E). CCK8 assay showed that the cell viability of the U87R and U251R cells was significantly suppressed by knowndown of TS (Figure 6F). More importantly, overexpression of TS with pTS dramatically abrogated the miR-203 induced re-sensitization to TMZ treatment (Figure 6G). To conclude, we demonstrated that miR-203 re-sensitizes the TMZ resistant GBM cells through directly targeting TS mRNA.

### MALAT1 inhibition reverses TMZ resistance by up-regulating miR-203 and suppressing TS expression

**Figure 7:**
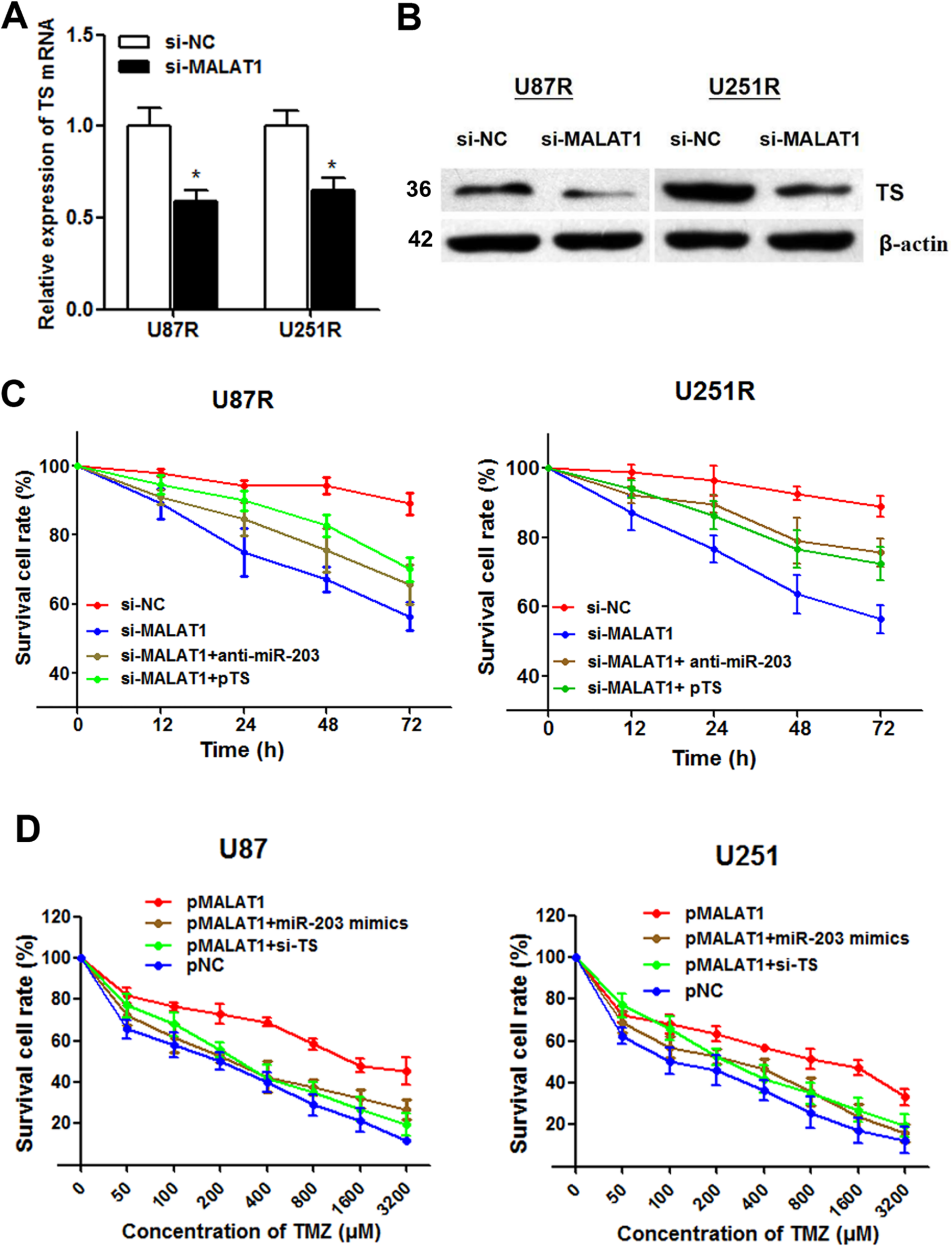
MALAT1 induces TMZ resistance through down-regulating miR-203 and promoting TS expression (**A**–**B**) MALAT1 knockdown significantly suppressed TS mRNA (A) and protein expression level (B) in U87R and U251R cells; (**C**) The increased cell cytotoxicity caused by MALAT1 knockdown was partially rescued by anti-miR-203 or pTS transfection in U87R and U251R cells; (**D**) The dose-effect curve showed that pMALAT1 transfection was followed by decreased cell death compared with the negative control, however, this increased TMZ resistance caused by MALAT1 was significantly reversed by miR-203 overexpression and TS inhibition. *P < 0.05.

After having found the interaction between miR-203 and TS, we doubt whether the function of MALAT1 is through miR-203-TS signaling pathway. MALAT1 knockdown significantly suppressed both TS mRNA and protein expression level in U87R and U251R cells (Figure 7A and 7B). Moreover, the increased cell cytotoxicity caused by MALAT1 knockdown was partially relieved by anti-miR-203 or pTS transfection (Figure 7C). This suggests that MALAT1 inhibition could reverse TMZ resistance by miR-203-TS pathway.

### MALAT1 induces TMZ resistance through down-regulating miR-203 and promoting TS expression

To directly validate that MALAT1 induces TMZ resistance in GBM cells, we treated parental U87 and U251 cells with a concentration gradient from 0–3200 μM TMZ after being transfected with pMALAT1 or negative control. The dose-effect curve showed that pMALAT1 significantly relieved the TMZ-induced cell cytotoxicity (Figure 7D). Besides, the IC_50_ values for TMZ were 4.60 and 5.87 times higher in U87 and U251 cells transfected with pMALAT1 than that in non-transfected cells, respectively. However, this increased TMZ resistance caused by MALAT1 was significantly reversed by miR-203 overexpression and TS inhibition (Figure 7D). Thus, we conclude that MALAT1 could partially induce TMZ resistance in GBM cells through miR-203-TS pathway.

### MALAT1 suppresses cell proliferation with G0/G1 cell cycle arrest in TMZ resistant cells

**Figure 8:**
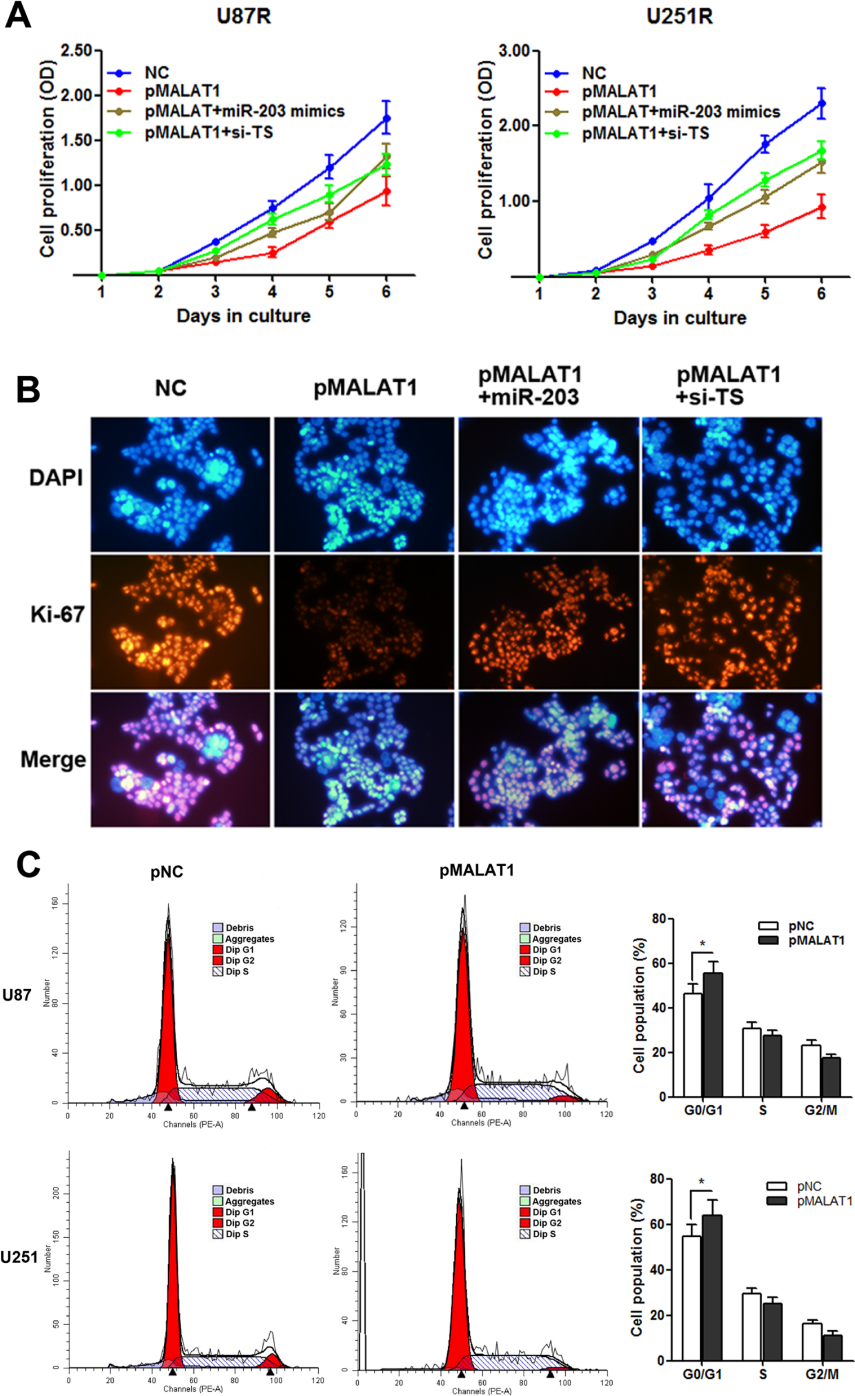
MALAT1 suppresses cell proliferation with G0/G1 cell cycle arrest in TMZ resistant cells (**A**) CCK8 assay indicated that pMALAT1 transfection significantly suppressed cell proliferation of U87R and U251R cells when the cells were incubated in the TMZ-free culture medium, however, this suppression was significantly rescued by co-transfection of miR-203 mimics or si-TS; (**B**) Decreased Ki-67 protein level was observed in U251R cells transefected with pMALAT1, and co-transfection of miR-203 or si-TS partially reversed the MALAT1 induced Ki-67 suppression by immunofluorescence analysis; (**C**) MALAT1 overexpression increased the percentage of G0/G1 cells and decreased the percentage of S-G2 cells in U87R and U251R cells. *P < 0.05.

It is known that TS is a critical regulator during cell proliferation [12, 13, 17], thus, we hypothesize that MALAT1 induces TMZ resistance of GBM through participating in the regulation of cell proliferation. As Figure 8A indicated, pMALAT1 transfection significantly suppressed cell proliferation of U87R and U251R cells when the cells were incubated in TMZ-free culture medium, however, this suppression was significantly rescued by co-transfection of miR-203 mimics or si-TS. Furthermore, the cell proliferation marker Ki-67 was detected by immunofluorescence analysis. Decreased Ki-67 expression was observed in U251R cells transefected with pMALAT1 when compared with negative control, and co-transfection of miR-203 or si-TS partially reversed the Ki-67 suppression (Figure 8B). Cell-cycle analysis indicated that MALAT1 significantly increased the percent of cell population in G0/G1 phase in U87R and U251R cells (Figure 8C). As decreased proliferative capacity is frequently observed in the chemo-resistant cancer cells, our results suggests that the MALAT1-miR-203-TS pathway participates TMZ resistance may through influencing the proliferation of GBM cell.

## DISCUSSION

Recently, it is urgent to find new molecules and mechanisms could shed light on the improved therapeutic strategies to overcome chemoresistance [18]. In currently study, high-throughput HiSeq sequencing was firstly performed to identify the candidate lncRNAs that may have diagnostic and prognostic value in GBM chemoresistance. Then the chosen lncRNAs were subjected to RT-qPCR assay to validate the deregulation. MALAT1 was finally identified to show high diagnostic value in discriminating responding patients from non-responding patients with considerable efficiency. Serum MALAT1 expression was associated with chemoresponse and predicted survival in GBM patients receiving TMZ treatment. More importantly, we demonstrated the molecular mechanism by which MALAT1 exerted its function in TMZ chemoresistance in GBM cells. We found that MALAT1 promoted cell proliferation and TMZ resistance through suppressing miR-203 expression and promoting TS expression.

LncRNA MALAT1 is located on Ch11q13 and has been reported to be deregulated and an independent prognostic parameter for survival in various cancers [19, 20]. It was firstly identified by Ji et al. as the lncRNA associated with metastasis in non-small cell lung cancer [21]. One study showed that MALAT1 associated with the malignant status and poor prognosis in glioma [22]. On the other hand, Han et al. revealed that MALAT1 was a tumor suppressor gene in glioma cells by downregulation of MMP2 and inactivation of ERK/MAPK signaling [23]. Thus, this contradictory conclusion indicates that more research is needed to investigate the functional role of MALAT1 in GBM. In our research, 10 candidate lncRNAs were firstly chosen to investigate their role in GBM chemoresistance. We tested the expression of the lncRNAs in both GBM tissues and serum, and we finally identified only one significantly altered lncRNA, MALAT1. Furthermore, ROC analysis confirmed that serum MALAT1 expression considerably distinguished responding patients from non-responding ones, with high AUC value. More importantly, further analyses showed that serum MALAT1 was correlated with the efficiency of chemotherapy. High expression of serum MALAT1 was associated with poor response to TMZ treatment in GBM patients. Additionally, high serum MALAT1 expression was associated with poor OS and RFS in patients receiving TMZ treatment. Based on these findings, we reveal that MALAT1 is a lncRNA highly expressed in non-responding GBM patients and closely correlated to chemoresponse to TMZ treatments.

Take a step further, we explored the underlying regulatory pathway that may account for the clinical findings. We found that MALAT1 expression was significantly increased in TMZ resistant cells compared with parental cells. Besides, MALAT1 knockdown dramatically promoted the cell cytotoxicity caused by TMZ. This date preliminary indicates that MALAT1 inhibition can reverse TMZ resistance in GBM cells. It is reported that lncRNAs may interacts with miRNAs [24]. Liu et al. showed that HOTAIR regulated EGFR-2 through competing with miR-331 [25]. Hirata et al. found that MALAT1 could promote renal cancer cell progression via interaction with miR-205 [26]. In contrast, another study indicated that miRNA-125b may suppressed lncRNA MALAT1 expression level in bladder cancer [27]. This suggest that interaction between MALAT1 and miRNAs may be a new regulatory mechanism. As expected, the computer algorithm miRcode identified two binding sites of miR-203 on MALAT1. Besides, our gain and loss function assay showed that MALAT1 suppressed miR-203 expression, a miRNA reported to play important roles during cancer progression and chemoresistance [28].

Various studies have shown that miR-203 played important roles during chemoresistance in cancer [29–31]. Our date showed that miR-203 mimics significantly abrogated TMZ resistance in U87R and U251R cells. It is known that miR-203 is known to induce cell growth delay and senescence, thus may function as a chemosensitization to cell cycle-specific drugs, such as oxaliplatin and paclitaxel. It was also reported to suppress chemoresistance in human glioblastoma by promoting epithelial-mesenchymal transition via SNAI2 [32]. Zhou et al. identified a mechanistic link of miR-203 to oxaliplatin resistance through ATM-mediated mechanism [33], which is consistent with the role of miR-203 in inducing cell-cycle arrest and senescence. Our results established TS mRNA as a direct target of miR-203 by using the luciferase experiments. Moreover, the function of miR-203 in TMZ resistance was partially relived when miR-203 mimics was co-transfected with pTS, suggesting that miR-203 exerts its regulatory role in TMZ resistance is through functional targeting of TS mRNA.

Based on the above observations, we hypothesize that MALAT1 promotes TMZ chemoresistance thorough miR-203-TS signaling pathway. As expected, MALAT1 knockdown significantly suppressed both TS mRNA and protein expression level in GBM cells. Moreover, the increased cell cytotoxicity caused by MALAT1 knockdown was partially relieved by anti-miR-203 or pTS transfection in U87R and U251R cells. On the other hand, enhanced MALAT1 expression significantly increased the IC_50_ values of TMZ and rescued the cell cytotoxicity caused by TMZ in U87 and U251 cells. This strongly confirmed that MALAT1 could promote TMZ resistance by miR-203-TS pathway in GBM.

Finally, we further investigated how the MALAT1-miR-203-TS pathway participated in TMZ resistance in GBM. As TS plays critical roles during the generation of thymidylate, TS may be a potential therapeutic target in GBM [17, 34]. Our results showed that MALAT1 suppressed cell proliferation through miR-203-TS pathway with G0/G1 cell cycle arrest in TMZ resistant cell lines. This date was further confirmed by the immunofluorescence analysis of proliferation maker, Ki-67. It is known that the characteristics of chem-resistant cells include enhanced migratory, invasive, and anti-apoptosis ability, in contrast to decreased cell proliferative capacity [35]. The decreased cell proliferation is helpful in promoting cancer cell viability when exposed to chemotherapy drugs [36]. To conclude, our results indicated that MALAT1 may promote GBM chemoresistance through influencing the pathway of cell proliferation.

In conclusion, our integrated approach demonstrates that MALAT1 expression is up-regulated in GBM patients who are resistant to TMZ treatment, and closely associated with chemoresponse status to TMZ treatment. We also reveal that MALAT1 can promote GBM chemoresistance and influence cell proliferation through suppressing miR-203 and promoting TS expression. These indicate that overexpression of MALAT1 confers a potent poor therapeutic efficacy. Thus, MALAT1 may be a potential prognostic marker and therapeutic target in GBM patients.

## MATERIALS AND METHODS

### Patients and samples

A multiphase, case–control study was designed to identify lncRNAs as potential biomarkers for differentiating chemo-response to TMZ therapy in GBM. Tumor response status was evaluated according to the Response Evaluation Criteria in Solid Tumors (RECIST) version 1.0 criteria and was assigned to patients with complete or partial response (CR and PR, respectively) and stable or progressive disease (SD and PD, respectively) in tumor measurements confirmed by repeat studies performed no less than four weeks after the criteria for response was first met. Briefly, 332 patients diagnosed with GBM but without other diseases were recruited from Sun Yat-Sen Memorial Hospital between January 2010 and February 2013. Among the 192 patients providing primary GBM tissues, there are 96 patients showing response (CR and PR) to TMZ treatment while the other 96 patients showing no response (SD and PD). All these participants were allocated to three phases. In the initial screening phase, tissue samples pooled from six patient showing response and six patients showing no response were subjected to Hiseq sequencing, to identify lncRNAs significantly differentially expressed. In the training phase, the candidate lncRNAs were tested with RT-qPCR in an independent cohort of primary tissues from 90 GBM patients responding to TMZ treatment and 90 patients showing no response to treatment. In the validation phase, another independent group of 140 GBM patients who provided serum samples were enrolled. Among these patients, there are 70 patients showing response to TMZ treatment while the other 70 patients showing no response.

All the patients were pathologically confirmed and the clinical tissue samples were collected before chemotherapy was started. They were classified according to the WHO criteria and staged according to the tumor-node-metastasis (TNM) classification. Fresh tumor tissue were immediately cut from the resected GBM tissue and kept at -80°C until RNA extraction. Venous blood was collected and centrifuged at 4000 rpm for 10 min, within 2 h. The supernatant fluids were then collected and further centrifuged at 12,000 rpm for 15 min to completely remove the cell debris. The whole process was strictly controlled to avoid hemolysis, and the supernatant serum was stored at −80°C, until analysis. Overall survival (OS) was updated on 1 February 2012 and was defined as the time from inclusion to death for any reason. Recurrence free survival (RFS) was defined as the time from inclusion to recurrence or metastasis progression. Written informed consent was obtained from all patients according to local ethical regulations of the Ethics Committee of Sun Yat-Sen Memorial Hospital.

### HiSeq sequencing

Total tissue RNA was extracted by one-step extraction using a Trizol kit (Life Technologies, USA), and the purity and quantity of RNA were determined by UV spectrophotometry. cDNA library construction and sequencing were performed according to previously described methods [37]. Briefly, after extraction of total RNA, ribosomal RNA was separated to isolate as ncRNA as possible. RNA containing poly(A) was then removed. RNA fragments were broken into short fragments randomly. The first chain of cDNA was generated using RNA fragments as templates and 6-bp random primers. Second chain of the cDNA was synthesized according to the kit's instruction (TakaRa Co., Ltd., Dalian, China). After purification, end repair, base A and sequencing joint adding, the generated cDNA was fragmented using uracil-N-glycosylase (UNG). cDNA fragments were chosen according to size, then PCR amplification was performed to establish the complete sequencing cDNA library. lncRNAs were sequenced using the high-throughput, high-sensitivity HiSeq 2500 sequencing platform (Illumina Company, USA). The whole process and subsequent data analysis were performed by Kangchen Bio-tech, Shanghai P.R. China. FastQC software was used for quality control of the pretreated data.

### Quantitative real-time PCR (RT-qPCR)

Total RNA was isolated from glioma specimens or glioma cell lines using TRIzol reagent (Invitrogen, Carlsbad, CA, USA). For lncRNAs and mRNA detection, the reverse transcription (RT) reactions were performed using a Prime Script™ RT Reagent Kit (Takara, Dalian, Liaoning). After mixing with 1 μg of template RNA, 4 μL of 5× Prime Script Buffer Mix, 1 μL of Prime Script RT Enzyme MixI, 1 μL of Oligo dT Primer and RNase-free dH2O in a final volume of 20 μL, the reaction volumes were incubated at 37°C for 30 min, followed by 85°C for 5 s and 4°C for 60 min. For real-time PCR, 2 μL of diluted generated cDNAs was mixed with 12.5 μL of SYBR Premix Ex TaqTM, 0.5 μL of DyeII, 1 μL forward and reverse primers (10 μM) and 9 μL of nuclease-free water in a final volume of 25 μL, according to the manufacturer's instructions (Takara Inc, Dalian). The reactions were incubated at 95°C for 30 s, followed by 45 cycles of 95°C for 5 s and 60°C for 34 s. Melting curve analysis was performed to evaluate the specificity of the RT-qPCR products. All reactions were run on CFX96™ real-time system (Bio-Rad, CA, American). Each RT-qPCR experiment was repeated three times. Relative expression of genes was calculated using the comparative cycle threshold (Ct) (2^−ΔΔCt^) method with glyceraldehyde-3-phosphate dehydrogenase (GAPDH) as the endogenous control.

For miRNA detection, the amounts of miRNAs were quantified in duplicate by (RT-qPCR) by using the human TaqMan MicroRNA Assay Kits (Applied Biosystems, Foster City, CA, USA). After the reverse transcription reaction which was carried out with TaqMan MicroRNA Reverse Transcription Kit (Applied Biosystems), cDNA solution was amplified using TaqMan Universal PCR Master MixII with no AmpErase UNG (Applied Biosystems). RT-PCR was run on 7500 Real Time PCR system (Applied Biosystems) with U6 as internal control. All reactions were performed in triplicate. The primer sequences are as follows (5′-3′): MALAT1 (forward): GGGTGTTTACGTAGACCAGAACC and MALAT1 (reverse): CTTCCAAAAGCCTTCTGCCTTAG; TS mRNA (forward): CTGCCAGCTGTACCAGAGAT and TS mRNA (reverse): ATGTGCATCTCCCAA AGTGT; GAPDH (forward): GCACCGTCAAGGCTGA GAAC and GAPDH (reverse): ATGGTGGTGAAGACGC CAGT; miR-203 (forward): ACACTCCAGCTGGGGT GAAATGTTTA and miR-203 (reverse): TGGTGTCGT GGAGTCG; U6 (forward): CTCGCTTCGGCAGCACA and U6 (reverse): AACGCTTCACGAATTTGCGT.

### Cell culture

The human U87 and U251 cell lines were purchased from the Cell Bank Type Culture Collection of the Chinese Academy of Sciences (Shanghai, China). Cells were grown in Dulbecco's modified Eagle's medium (DMEM) (Hyclone; Thermo Fisher Scientific, Waltham, MA, USA) supplemented with 10% fetal bovine serum (FBS) (Gibco, Invitrogen Inc., Carlsbad, CA, USA), 100 U/ml penicillin and 100 g/ml streptomycin (Life Technologies, Grand Island, NY, USA) at 37°C in 5% CO2 and 95% air. The cell authenticity was determined by short tandem repeat analysis technology (Cell IDTM System, Promega, Madison, WI).

### Development of TMZ-resistant cell lines

TMZ was purchased from Sigma-Aldrich (St. Louis, MO, USA) and diluted in Dimethyl sulfoxide (Solorbio Inc, Beijing, China) to a stock solution of 200 mM TMZ. U87 and U251 cells were exposed to an initial TMZ concentration of 10 μmol/L in DMEM plus 10% FBS. The surviving population of cells was grown to 80% confluence and passaged twice over 9 days to ensure viability. The concentration of TMZ that the surviving population was exposed to was then sequentially increased in the same manner to 50 μmol/L (15days), 100 μmol/L (30 days), 200μmol/L (50 days) and finally to the clinically relevant plasma concentration of 325 μmol/L [38, 39]. For all *in vitro* studies, TMZ-resistant cells was used at no higher than 15 passages from creation and were maintained and exposed to 325 μM TMZ unless otherwise indicated.

### RNA oligoribonucleotides and cell transfection

The small interfering RNAs (siRNAs) that specifically target human MALAT1, miR-203 and TS mRNA were designated as si-MALAT1, anti-miR-203 and si-TS, respectively. The MALAT1 overexpression plasmid (pMALAT1) was purchased from Addgene. The coding sequence of TS was amplified and then cloned into PCDNA3.1 vector, and was named as pTS. The negative control duplex (NC) for both miRNA mimics and siRNA, as well as the single standard negative control RNA for miRNA inhibitors (anti-NC), was not homologous to any human genome sequences. All RNA oligoribonucleotides were purchased from RiboBio (Guangzhou, China). The transfection of RNA oligoribonucleotides (100 nmol/L) and plasmid was performed by using Lipofectamine 2000 (Invitrogen).

### Cell viability assay

Cell viability was quantified by using the Cell Counting Kit-8 (CCK-8; Beyotime). Briefly, 100 μl of cells from the different transfection group were seeded onto a 96-well plate at a concentration of 2000 cells per well and were incubated at 37°C. At different time point, the optical density was measured at 450 nm using a microtiter plate reader, and the rate of cell survival was expressed as the absorbance. The results represent the mean of three replicates under the same conditions.

### Cell cycle analysis

After transfection, cells were washed in PBS and fixed in 70% ethanol at 4°C for 2 hours. DNA staining was done with 10 mg propidium iodide/mL PBS and 2.5 Ag DNase-free RNase (Roche Diagnostics)/mL PBS for at least 30 minutes before flow cytometry in a Coulter EPICS XL flow cytometer (Beckman Coulter, Inc., Fullerton, CA). Cell cycle profiles were generated from flow cytometry analysis with Modifit software (BD Biosciences).

### Dual-luciferase reporter assay

The putative miR-203 binding sites in the TS 3′-UTR was predicted by TargetScan and miRanda. Dual-luciferase reporter assay was performed using pmiR-REPORTTM vectors (RiboBio, Guangzhou, China) containing wild-type TS 3′-UTR sequences or mutant TS 3′-UTR sequences. Cells (1×10^5^) were transiently transfected with miR-203 mimics or negative control together with wild-type TS 3′-UTR vector or mutant type TS 3′-UTR vector in a 24-well plate. Cells were harvested 48 h after transfection, and luciferase activity was analysed by the Dual-luciferase Reporter Assay Kit (Promega) according to the manufacturer's instructions.

### Western blot and antibodies

The primary antibodies used for western blotting were rabbit anti-human TS antibody (1:1000; Novas) and rabbit anti-human β-actin antibody (1:1000; Cell Signaling Technology). Horseradish peroxidase-conjugated (HRP) anti-rabbit antibodies (1:5000; Santa Cruz Biotechnology) were used as the secondary antibodies. A total of 25 μg protein from each sample was separated on 10% Bis-Tris polyacrylamide gel through electrophoresis and then blotted onto polyvinylidene fluoride (PVDF) membranes (GE Healthcare, Piscataway, NJ, USA). Blots were immunostained with primary antibody at 4°C overnight and with secondary antibody at room temperature for 1 h. Immunoblots were visualised by using Immobilon^TM^ Western Chemiluminescent HRP Substrate (Millipore). Protein levels were normalized to β-actin.

### Immunofluorescence analysis

U87 cells were grown to 40% to 50% confluence and then transfected with 100 nM of pMALAT1, miR-203 mimics or si-TS. After 48 hours of incubation, the cells were fixed with 4% paraformaldehyde and permeabilized in 0.2% Triton X-100 (Sigma-Aldrich) for 20 minutes. The cells were then blocked with 10% goat serum in PBS for 1 h. Cells were incubated with primary anti-Ki-67 (Cell Signaling Technology) overnight at 4°C and then incubated with the appropriate rhodamine-conjugated secondary antibody for 1 h. The cells were then washed and incubated with DAPI (Invitrogen) for nuclear staining. The slides were visualized for immunofluorescence with a laser scanning Olympus microscope.

### Statistical analysis

Kolmogorov-Smirnov test was used to determine the normality of the distribution of data in each group. Data were presented as median (interquartile range). Difference of lncRNA levels among multiple groups in Hiseq sequencing was determined by Bonferroni adjustment method. Mann-Whitney U test or Kruskal-Wallis test was employed to compare differences of lncRNAs or miRNAs among clinical cohort groups. Count dates were described as frequency and examined using Fisher's exact test. Receiver operating characteristic (ROC) curves were established to discriminate GBM responding patients from non-responding patients. Area under the ROC curve (AUC) was used as an accuracy index for evaluating the predictive performance of suspected lncRNA [40]. The Youden index (sensitivity + specificity -1) was used to determine the optimal cutoff point [41]. Survival curves were estimated with Kaplan-Meier method and comparisons were conducted using log-rank test. For comparison of different GBM cell lines and different GBM tissue groups, lncRNA or miRNA expression levels and luciferase reporter assays were treated as continuous variables, and differences in mean expression were determined using Student's *t*-test. Correlation testing between serum MALAT1 and miR-203 expression was analysed using Spearman test. Differences in cell growth curves and cell cytotoxicity curves were determined by repeated measures analysis of variance. Cox proportional hazards regression model was employed to identify independent prognostic factors. Survival curves were drawn by using SPSS 19.0 (SPSS lnc., Chicago, IL., USA). MedCalc 9.3.9.0 (MedCalc, Mariakerke, Belgium) was used for ROC analysis, and other analyses were performed with GraphPad Prism 5.0 (GraphPad Software, La Jolla, California, USA). Error bars in figures represent SD (Standard Deviation), and statistical significance was defined as two-sided *P* value < 0.05.
